# Supplementary material for: Darwin's vexing contrivance: a new hypothesis for why some flowers have two kinds of anther
Source: Proc Biol Sci. 2020 Dec 23;287(1941):20202593. doi: 10.1098/rspb.2020.2593 (PMC7779490; doi:10.1098/rspb.2020.2593)
Supplement: Supplementary Table, Figures, Appendix, and References [file rspb20202593supp1.pdf]

Electronic Supplementary Material for

Darwin’s vexing contrivance: a new hypothesis for why some flowers have two kinds of anthers

Kathleen M. Kay*, Tania Jogesh, Diana Tataru, Sami Akiba

Proceedings of the Royal Society B

DOI: 10.1098/rspb.2020.2593

*Email: kmkay@ucsc.edu

This file includes:

Figures S1 to S4
Table S1
Legend for Movie S1
Appendix S1
SI References

Other supplementary files for this manuscript include the following:

Movie S1

All data and code for this manuscript can be found at:

https://doi.org/10.5061/dryad.8cz8w9gmp

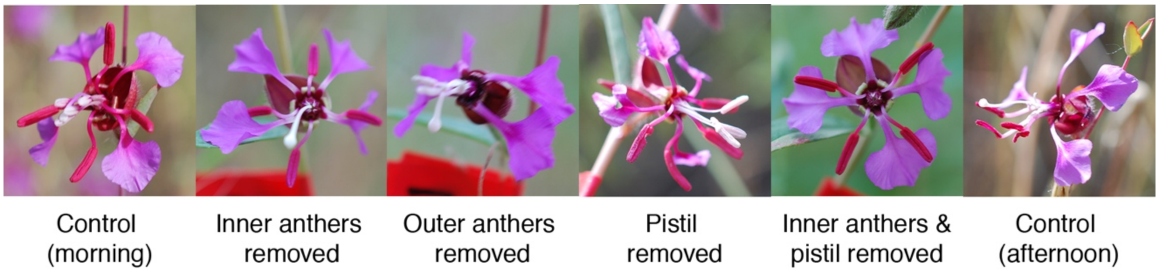

Fig. S1. Representative photos of the treatments in our *C. unguiculata* anther removal field experiment.

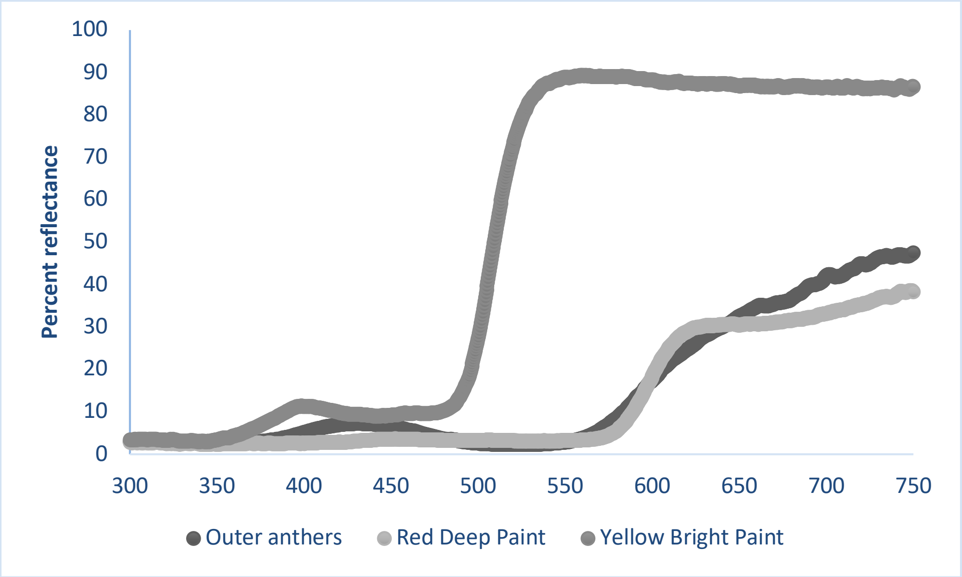

Fig. S2. Colour reflectance of the paint used in the *C. unguiculata* anther colour manipulation experiment. Reflectance of the outer anthers is shown for comparison. Red Deep paint was chosen as a control that approximately matched the colour of the outer anthers. Yellow Bright was chosen to be highly visible to bees. Reflectance measures were taken with an Ocean Optics JAZ spectrometer, with a full spectrum light source (Ocean Optics PX-2, pulsed xenon lamp 220-750 nm) and the following settings: integration time of 10 milliseconds, scans to average = 50, and boxcar width = 5. Reflectance readings were scaled to a white standard (Ocean Optics WS-1-SL Spectralon Reflectance Standard, 99% reflectivity 250-2500 nm). All paint was Blick Matte Acrylic.

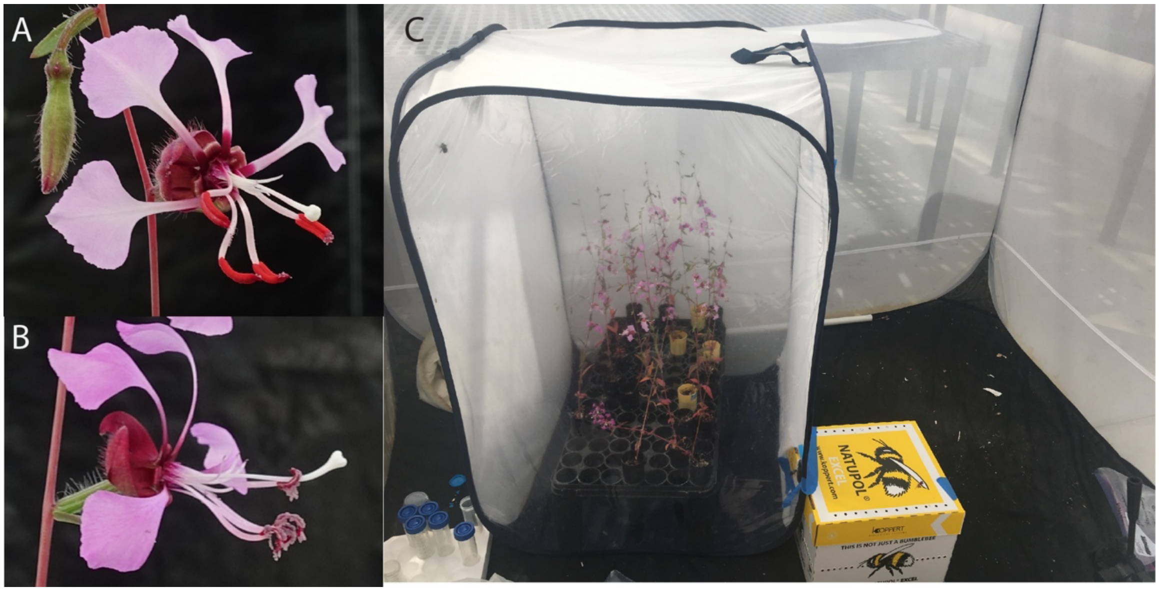

Fig. S3. Manipulations of the pollen release schedule of *Clarkia unguiculata*. A & B: Representative examples of “dosing” and “no dosing” flowers, respectively. Inner anthers have been removed from both to isolate reponses to pollen release schedules of the outer anthers. C. Our experimental set up showing the *Bombus impatiens* hive directly connected to the cage containing the flower array. Arrays comprised 20 male phase flowers and 5 female phase flowers. Each array was exposed to a single bee pollinator, and once the bee began visiting flowers, we unzipped the small cage to allow the bee to freely leave the array.

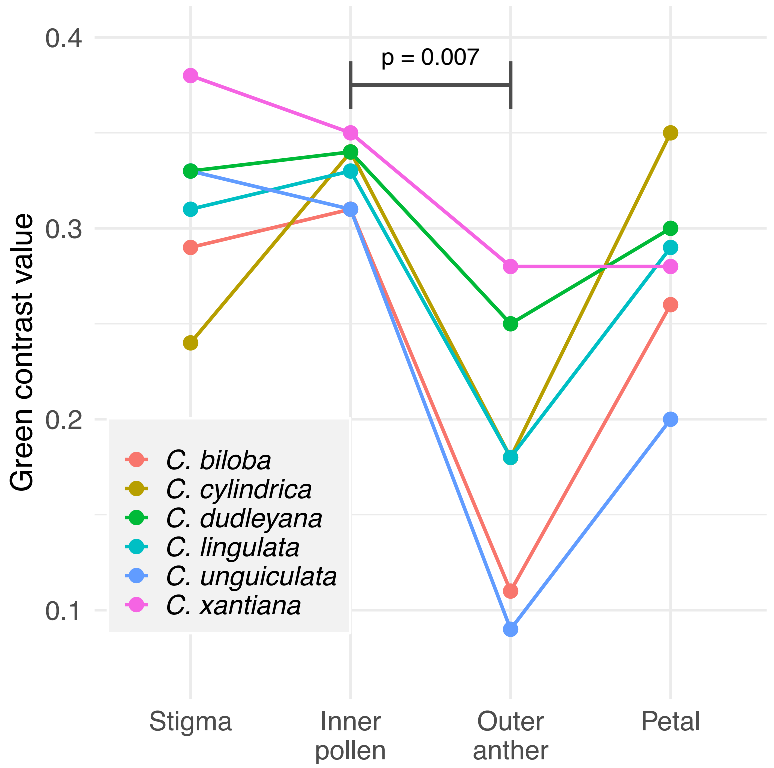

Fig. S4. Green contrast values from flower colour reflectance from six heterantherous *Clarkia* species. In freshly opened flowers, undehisced outer anthers are significantly less conspicuous than the exposed pollen from inner anthers.

Table S1. Clarkia taxa, character states, and references for the comparative study of heteranthery.							
Taxa	Heteranthery	Binary pollination	Pollination	Pollination citation	Anther movement	Anther timing separation index	Collector number; herbarium
C. amoena ssp. *amoena*	no	Bee	Bee, beetle	(1)	no	NA	Kay 715; UCSC
C. arcuata	no	Other	Autogamous	(2)	no	NA	Gottlieb 15877; UCSC
C. biloba ssp. *biloba*	yes	Bee	Bee	(3)	yes	0.902	Grossenbacher 1099; UCSC
C. bottae	yes	Bee	Bee	(1)	yes	NA	Weeden 35-4; DAV
C. breweri	no	Other	Hawkmoth	(4)	no	0.000	B. O'Brien 16827; UCSC
C. concinna ssp. *concinna*	no	Other	Fly, bee, butterfly	(4)	no	0.000	Gottlieb 15884; UCSC
C. cylindrica ssp. *cylindrica*	yes	Bee	Bee	(1)	yes	0.797	Wilsura & Hayduk 17739; UCSC
C. dudleyana	yes	Bee	Bee	(1)	yes	0.794	Wilsura 21170; UCSC
C. epilobioides	no	Other	Autogamous	(2)	no	0.000	Wall 21078; UCSC
C. franciscana	no	Other	Autogamous	(5)	no	NA	Gottlieb F28-2-2; DAV
C. heterandra	no	Other	Autogamous	(2)	no	NA	Weeden 6; DAV

C. imbricata	no	Bee	Bee	(1)	no	NA	Gottlieb PG-1 DAV
C. lewisii	yes	Bee	Bee	(1)	yes	NA	Weeden 35-4; DAV
C. lingulata	yes	Bee	Bee	(6)	yes	0.466	Grossenbacher 1098; UCSC
C. mildrediae ssp. *mildrediae*	no	Bee	Bee	(2)	yes	NA	Weeden 50; DAV
C. modesta	yes	Other	Autogamous	(2)	no	NA	NA
C. rostrata	yes	Bee	Bee	(1)	yes	NA	Weeden 97a; DAV
C. rubicunda	no	Bee	Bee	(1)	no	0.000	Kay 718; UCSC
C. speciosa ssp. *speciosa*	no	Bee	Bee	(7)	no	NA	Kay 602; UCSC
C. unguiculata	yes	Bee	Bee	(1)	yes	0.875	Kay 603; UCSC
C. virgata	no	Bee	Bee	(1)	yes	NA	Gottlieb 15910; UCSC
C. williamsonii	no	Bee	Bee	(1, 7)	no	NA	Gottlieb 15913; UCSC
C. xantiana ssp. *xantiana*	yes	Bee	Bee	(8, 9)	yes	0.991	Gottlieb 7438; UCSC

Movie S1 (separate file). Time-lapse movie of *Clarkia unguiculata* flower development.

We took photos every 10 minutes with a digital SLR camera and interval timer, continually adjusting the focus over the course of anthesis**.** To make time-lapse movies of each flower, we imported photos into Adobe Lightroom, and adjusted temperature, tint, exposure, clarity, and vibrance to make photo sequences somewhat consistent. We then exported sequenced photos to Adobe Photoshop, converted them to JPEG, and rendered video at 10 frames per second using H.264 compression.

Appendix S1. Statistical models and results

a. Correlated evolution between color heteranthery and other traits

We used Pagel's maximum likelihood test of correlated evolution between color heteranthery (two colours of anthers v. one colour of anthers) in the R package phytools and compared the fit of a series of models. We used the fitmk method of maximum likelihood optimization in which transitions among character states follow a Markov process, and we used the ARD setting in which all transition rates can be different. For each model, we specified whether one transition rate serves as a dependent variable while the state of the other character serves as an explanatory variable ("change in x depends on y state"), whether the transition rates of both characters vary independently of the state of the other character, or whether the transition rates of both characters depend on the state of the other character.

Pagel's test of correlated evolution between pollination (bee v. other) and colour heteranthery (yes or no)

**Tested model**	**Likelihood ratio p-value (tested against independence)**	**AIC**
Change in heteranthery depends on pollination state	0.055	53.58845
Transition rates are independent of the other character state	NA	55.3752
Change in pollination depends on heteranthery state	0.16	55.72302
Change in both characters depends on the other character state	0.21	57.48688

Pagel's test of correlated evolution between anther movement (yes or no) and colour heteranthery (yes or no)

**Tested model**	**Likelihood ratio p-value (tested against independence)**	**AIC**
Change in heteranthery depends on anther movement state	0.004	44.72701
Transition rates are independent of the other character state	NA	51.70650
Change in movement depends on heteranthery state	0.020	47.88305
Change in both characters depends on the other character state	0.015	47.30821

b. Pollen deposition on *C. cylindrica* stigmas and bee pollinators

We used a linear model with a fixed factor ("Indicator") of the sample source (bee scopae v. plant stigmas). The response variable ("prop_purple" is the proportion of the total sample made up of purple pollen from the outer anthers.

lm(formula = prop_purple ~ Indicator + total_pollen, data = data_subset)

Residuals:

Min	1Q	Median	3Q	Max
-0.34244	-0.10400	-0.00535	0.08745	0.52455

Coefficients:

Estimate	Std.	Error	t value	Pr(>|t|)
(Intercept)	2.453e-01	5.486e-02	4.472	6.81e-05 ***
IndicatorStigma	-8.438e-02	6.021e-02	-1.401	0.16922
total_pollen	1.943e-04	6.506e-05	2.986	0.00493 **

Signif. codes: 0 '***' 0.001 '**' 0.01 '*' 0.05 '.' 0.1 ' ' 1

Residual standard error: 0.1823 on 38 degrees of freedom
Multiple R-squared: 0.2838, Adjusted R-squared: 0.2461
F-statistic: 7.527 on 2 and 38 DF, p-value: 0.001763

We also examined a model with an interaction term between Indicator and total_pollen, but it did not have additional explanatory power and the interaction term was not significant. Thus, the proportion of purple pollen increases with the number of pollen grains similarly regardless of whether the sample comes from the bee scopa or the stigma.

lm(formula = prop_purple ~ Indicator + total_pollen + Indicator * total_pollen, data = data_subset)

Residuals:

Min	1Q	Median	3Q	Max
-0.34225	-0.09281	-0.01024	0.08969	0.52383

Coefficients:

Estimate	Std.	Error	t value	Pr(>|t|)
(Intercept)	2.430e-01	5.588e-02	4.349	0.000103 ***
IndicatorStigma	-5.059e-02	1.152e-01	-0.439	0.663260
total_pollen	1.984e-04	6.691e-05	2.965	0.005268 **
IndicatorStigma:total_pollen	-1.295e-04	3.748e-04	-0.345	0.731734

Signif. codes: 0 '***' 0.001 '**' 0.01 '*' 0.05 '.' 0.1 ' ' 1

Residual standard error: 0.1845 on 37 degrees of freedom
Multiple R-squared: 0.2861, Adjusted R-squared: 0.2282
F-statistic: 4.942 on 3 and 37 DF, p-value: 0.005511

c. Field manipulations of *Clarkia unguiculata* flowers

We used generalized linear mixed models with Poisson error distributions to assess how bees responded to manipulated *C. unguiculata* flowers. Flower treatment was a fixed effect, and flower patch was a random effect. We analyzed morning and afternoon data separately. In the morning, outer anthers were closed and reflexed, whereas inner anthers had available pollen. In the afternoon, inner anthers were generally stripped bare, and outer anthers were erect and had available pollen.

Full model results for the morning:

Generalized linear mixed model fit by maximum likelihood (Laplace Approximation) ['glmerMod']
Family: poisson (log)
Formula: Total_visits ~ Treatment + (1 | Patch)
Data: cungMorning

AIC	BIC	logLik	deviance	df.resid
359.9	375.2	-174.0	347.9	89

Scaled residuals:

Min	1Q	Median	3Q	Max
-2.2383	-0.7845	-0.3389	0.6800	4.0872

Random effects:

Groups Name	Variance	Std.Dev.
Patch (Intercept)	0.8537	0.9239

Number of obs: 95, groups: Patch, 17

Fixed effects:

	Estimate	Std. Error	z value	Pr(>|z|)
(Intercept)	0.5800	0.2810	2.064	0.03903 *
Treatment2	-0.6737	0.2370	-2.843	0.00447 **
Treatment3	-0.1942	0.2049	-0.948	0.34336
Treatment4	-0.2942	0.2107	-1.396	0.16264
Treatment5	-1.2238	0.2888	-4.237	2.27e-05 ***

Treatment order is as presented in Figures 5 and S1, with the control serving as Treatment 1.

Signif. codes: 0 ‘***’ 0.001 ‘**’ 0.01 ‘*’ 0.05 ‘.’ 0.1 ‘ ’ 1

Confidence intervals

	2.5 %	97.5 %
.sig01	0.56419086	1.5702964
(Intercept)	-0.06854635	1.1161603
Treatment2	-1.16046250	-0.2112554
Treatment3	-0.60670243	0.2129869
Treatment4	-0.72015537	0.1230474
Treatment5	-1.83423632	-0.6740561

Full model results for the afternoon:

Generalized linear mixed model fit by maximum likelihood (Laplace Approximation) ['glmerMod']
Family: poisson (log)
Formula: Total_visits ~ Treatment + (1 | Patch)
Data: cungAfternoon

AIC	BIC	logLik	deviance	df.resid
401.6	415.9	-194.8	389.6	74

Scaled residuals:

Min	1Q	Median	3Q	Max
-2.3008	-0.8977	-0.3333	0.8291	3.2742

Random effects:

Groups Name	Variance	Std.Dev.
Patch (Intercept)	0.6168	0.7854

Number of obs: 80, groups: Patch, 16

Fixed effects:

	Estimate	Std. Error	z value	Pr(>|z|)
(Intercept)	1.4724	0.2273	6.478	9.3e-11 ***
Treatment2	-0.3171	0.1590	-1.994	0.046139 *
Treatment3	-0.5705	0.1718	-3.322	0.000895 ***
Treatment4	-0.5145	0.1688	-3.049	0.002299 **
Treatment5	-0.6506	0.1763	-3.690	0.000224 ***

Treatment order is as presented in Figures 5 and S1, with the control serving as Treatment 1.

Signif. codes: 0 ‘***’ 0.001 ‘**’ 0.01 ‘*’ 0.05 ‘.’ 0.1 ‘ ’ 1

> CungAfternoon_CI

	2.5 %	97.5 %
.sig01	0.5324386	1.226585303
(Intercept)	0.9849202	1.925054454
Treatment2	-0.6351425	-0.004256229
Treatment3	-0.9167981	-0.235029153
Treatment4	-0.8540108	-0.184289807
Treatment5	-1.0068740	-0.307094093

d. Bumblebee trials in the greenhouse with *Clarkia unguiculata* flowers that either have a small amount of pollen available or all their pollen available (i.e., “dosing” v. “no dosing” flowers)

We compared the pollen export to stigmas between dosing and no dosing flowers using a generalized linear mixed model with treatment as a fixed effect and the unique flower array/bee combination as a random effect.

Generalized linear mixed model fit by maximum likelihood (Laplace Approximation) ['glmerMod']
Family: Negative Binomial(0.3711) (log)
Formula: Pollen ~ Treatment + (1 | Date_array)
Data: stigmas

AIC	BIC	logLik	deviance	df.resid
1139.8	1151.4	-565.9	1131.8	132

Scaled residuals:

Min	1Q	Median	3Q	Max
-0.6062	-0.5771	-0.4050	0.0960	4.6168

Random effects:

Groups Name	Variance	Std.Dev.
Date_array (Intercept)	0.1001	0.3164

Number of obs: 136, groups: Date_array, 27

Fixed effects:

	Estimate	Std. Error	z value	Pr(>|z|)
(Intercept)	3.5911	0.2645	13.576	<2e-16 ***
TreatmentNo dosing	-0.3507	0.3183	-1.102	0.27

--Signif. codes: 0 ‘***’ 0.001 ‘**’ 0.01 ‘*’ 0.05 ‘.’ 0.1 ‘ ’ 1

SI References

1. J. MacSwain, P. H. Raven, R. Thorp, Comparative behavior of bees and Onagraceae. IV. *Clarkia* bees of the western United States. *University of California Publications in Entomology* **70**, 1–80 (1973).

2. H. Lewis, M. E. Lewis, *The genus* Clarkia (University of California Press, 1955).

3. H. Lewis, M. R. Roberts, The origin of *Clarkia lingulata*. *Evolution* **10**, 126–138 (1956).

4. T. J. Miller, R. A. Raguso, K. M. Kay, Novel adaptation to hawkmoth pollinators in *Clarkia* reduces efficiency, not attraction of diurnal visitors. *Annals of Botany* **113**, 317–329 (2014).

5. L. D. Gottlieb, Enzyme differentiation and phylogeny in *Clarkia franciscana*, *Clarkia rubicunda* and *Clarkia amoena*. *Evolution* **27**, 205–214 (1973).

6. H. Lewis, Catastrophic selection as a factor in speciation. *Evolution* **16**, 257–271 (1962).

7. W. E. LaBerge, R. W. Thorp, A revision of the bees of the genus *Andrena* of the Western Hemisphere. Part XIV—Subgenus Onagrandrena (2005) (May 26, 2020).

8. V. Eckhart, N. Rushing, G. Hart, J. Hansen, Frequency-dependent pollinator foraging in polymorphic *Clarkia xantiana* ssp. *xantiana* populations: implications for flower colour evolution and pollinator interactions. *Oikos* **112**, 412–421 (2006).

9. D. A. Moeller, Pollinator community structure and sources of spatial variation in plant-pollinator interactions in *Clarkia xantiana* ssp. *xantiana*. *Oecologia* **142**, 28–37 (2005).
